# Development a novel robust method to enhance the solubility of Oxaprozin as nonsteroidal anti-inflammatory drug based on machine-learning

**DOI:** 10.1038/s41598-022-17440-4

**Published:** 2022-07-30

**Authors:** Walid Kamal Abdelbasset, Safaa M. Elkholi, Khadiga Ahmed Ismail, Sameer Alshehri, Ahmed Alobaida, Bader Huwaimel, Ahmed D. Alatawi, Amal M. Alsubaiyel, Kumar Venkatesan, Mohammed A. S. Abourehab

**Affiliations:** 1 Department of Health and Rehabilitation Sciences, College of Applied Medical Sciences, Prince Sattam Bin Abdulaziz University, P.O. Box. 173, Al-Kharj, 11942 Saudi Arabia; 2 Department of Physical Therapy, Kasr Al-Aini Hospital, Cairo University, Giza, 12613 Egypt; 3 Department of Rehabilitation Sciences, College of Health and Rehabilitation Sciences, Princess Nourah Bint Abdulrahman University, P.O. Box. 84428, 11671 Riyadh, Saudi Arabia; 4 Department of Clinical Laboratory Sciences, College of Applied Medical Sciences, Taif University, P.O.BOX.11099, Taif, 21944 Saudi Arabia; 5 Department of Pharmaceutics and Industrial Pharmacy, College of Pharmacy, Taif University, P.O.Box 11099, Taif, 21944 Saudi Arabia; 6 Department of Pharmaceutics, College of Pharmacy, University of Hail, Hail, 81442 Saudi Arabia; 7 Department of Pharmaceutical Chemistry, College of Pharmacy, University of Hail, Hail, 81442 Saudi Arabia; 8 Department of Clinical Pharmacy, College of Pharmacy, Jouf University, Sakaka, Al-Jouf Saudi Arabia; 9 Department of Pharmaceutics, College of Pharmacy, Qassim University, Buraidah, 52571 Saudi Arabia; 10 Department of Pharmaceutical Chemistry, College of Pharmacy, King Khalid University, Abha, 62529 Kingdom of Saudi Arabia; 11 Department of Pharmaceutics, College of Pharmacy, Umm Al-Qura University, Makkah, 21955 Saudi Arabia; 12 Department of Pharmaceutics and Industrial Pharmacy, Faculty of Pharmacy, Minia University, Minia, 61519 Egypt

**Keywords:** Green chemistry, Chemistry, Drug delivery

## Abstract

Accurate specification of the drugs’ solubility is known as an important activity to appropriately manage the supercritical impregnation process. Over the last decades, the application of supercritical fluids (SCFs), mainly CO_2_, has found great interest as a promising solution to dominate the limitations of traditional methods including high toxicity, difficulty of control, high expense and low stability. Oxaprozin is an efficient off-patent nonsteroidal anti-inflammatory drug (NSAID), which is being extensively used for the pain management of patients suffering from chronic musculoskeletal disorders such as rheumatoid arthritis. In this paper, the prominent purpose of the authors is to predict and consequently optimize the solubility of Oxaprozin inside the CO_2_SCF. To do this, the authors employed two basic models and improved them with the Adaboost ensemble method. The base models include Gaussian process regression (GPR) and decision tree (DT). We optimized and evaluated the hyper-parameters of them using standard metrics. Boosted DT has an MAE error rate, an R2-score, and an MAPE of 6.806E-05, 0.980, and 4.511E-01, respectively. Also, boosted GPR has an R2-score of 0.998 and its MAPE error is 3.929E-02, and with MAE it has an error rate of 5.024E-06. So, boosted GPR was chosen as the best model, and the best values were: (T = 3.38E + 02, P = 4.0E + 02, Solubility = 0.001241).

## Introduction

Recently, numerous endeavors have been made to find various efficacious and promising techniques to appropriately solve or at least mitigate the unprecedented challenges of pharmaceutical industry such as low solubility of drugs, unacceptable productivity and growing research and development (R&D) costs^1–3^. Oxaprozin (known under the brand name Daypro) is a well-known nonsteroidal anti-inflammatory drug (NSAID), which has indication in pain/swelling management of adult patients suffering from rheumatoid arthritis, ankylosing spondylitis and soft tissue disorders^4^. Microsomal oxidation and glucuronic acid conjugation are known as the major procedures of Oxaprozin primary metabolism in the liver. Metabolism of this drug in the liver results in the formation of Ester and ether glucuronides as the prominent conjugated metabolites. Manageable safety profile, great efficiency, low liver toxicity and appropriate cost has made Oxaprozin a golden NSAID for the pain alleviation of patients with chronic musculoskeletal diseases^5–7^.

Application of novel approaches to increase the poor solubility of drugs is an attractive approach to solve one of the challenges of pharmaceutical industry. Recently, the use of supercritical fluids (SCFs) for processing therapeutic agents has offered suitable opportunities for the pharmaceutical manufacturing scientists^8^. This type of fluid possesses great potential of application in disparate scientific scopes including drug delivery, chromatography, and extraction^9^. Among various sorts of SCFs, supercritical carbon dioxide (SCCO_2_) recommends various interesting technological advantages such as low toxicity, ignorable flammability and environmentally friendly characteristic which may eventuate in result a significant decrement in the application of commonly employed organic solvents. Apart from different industrial-based applications, particle micronization using SCCO_2_ is one of the novel and promising approaches for fabricating micro-/nanoparticles with controlled size and purity^10^.

Prediction of drugs solubility using artificial intelligence (AI) method has currently attracted the attention as a noteworthy option for validating the actual data obtained from experimental research. Development of predictive modeling and simulation via this technique for different industries (i.e., separation, purification, extraction and drug delivery) can considerably decline computation time and guarantee the accuracy of conducted experimental results^11,12^.

Computers can learn from data without having to be explicitly programmed, using a class of AI techniques known as machine learning (ML). Machine learning seeks to develop meta-programs that process experimentally gathered data and apply it to train models for the prediction of unknown future inputs^13,14^. Ensemble methods are also a class of ML methods that use several basic models to achieve higher accuracy and generality in prediction^15,16^.

When multiple weak estimators are combined to produce a robust estimator, it is known as "boosting." Because of the sequential logic employed by Boosting, each weak estimator has a direct impact on its successor. Particularly AdaBoost^17^ is a typical boosting algorithm that uses reweighted training data to gradually obtain weak classifiers. It was decided to use Adaboost procedures to modify the efficiency of two base estimators as the foundation of this study. Decision Tree and Gaussian process regression are selected base models.

Decision Tree asks a series of questions using feature sets, such as ‘is equal' or ‘is greater,' and based on the provided answers, another question is asked to respond. Same procedure is repeated until no further inquiries are received, at the point the result is obtained. The data is constantly divided into binary components, allowing the Decision Tree to grow. To evaluate the divisions for all attributes, a randomness metric such as entropy is used^18,19^.

Also, for both exploration and exploitation, Gaussian process regression is a non-parametric Bayesian modeling technique. The primary profitability of the method is the ability to forge a reliable response for input variables. It can describe a broad range of interactions between features and targets by using a feasibly infinite count of input features and allowing the data to define the complexity level through Bayesian inference^20,21^.

## Experimental

In this paper, validation of predictive models’ results is done by their comparison with obtained experimental data from the experiments of Khoshmaram et al.^22^. They developed a pressure-volume-temperature (PVT) cell to experimentally measure the solubility value of Oxaprozin in SCCO_2_ solvent^22^. In their developed setup, first, the SCCO_2_ solvent is prepared via increasing the pressure of gaseous CO_2_ through the liquefaction unit. In the second step, the impurities of condensed manufactured SCOO_2_ are removed via an inline filter. Then after, the purified SCOO_2_ flows through a surge tank before its entrance to the PVT cell. The controlling process of temperature as an important parameter directly affects the solubility value of drug takes place using heating elements that wrapped the chamber and are isolated via PTFE layer.

## Data Set

The dataset used in this study comes from^22^, which has just 32 data points. The temperature and pressure are two input parameters. Each vector also has one output (solubility). Table 1 shows the dataset.

**Table 1:** The Whole Dataset.

No	Temperature (K)	Pressure (bar)	Solubility (mole fraction)
1	3.08E + 02	1.20E + 02	8.19E-05
2	3.08E + 02	1.60E + 02	1.58E-04
3	3.08E + 02	2.00E + 02	2.24E-04
4	3.08E + 02	2.40E + 02	2.80E-04
5	3.08E + 02	2.80E + 02	3.44E-04
6	3.08E + 02	3.20E + 02	4.06E-04
7	3.08E + 02	3.60E + 02	4.73E-04
8	3.08E + 02	4.00E + 02	5.33E-04
9	3.18E + 02	1.20E + 02	7.55E-05
10	3.18E + 02	1.60E + 02	1.41E-04
11	3.18E + 02	2.00E + 02	2.45E-04
12	3.18E + 02	2.40E + 02	3.56E-04
13	3.18E + 02	2.80E + 02	4.64E-04
14	3.18E + 02	3.20E + 02	5.58E-04
15	3.18E + 02	3.60E + 02	6.60E-04
16	3.18E + 02	4.00E + 02	7.66E-04
17	3.28E + 02	1.20E + 02	5.34E-05
18	3.28E + 02	1.60E + 02	1.28E-04
19	3.28E + 02	2.00E + 02	3.02E-04
20	3.28E + 02	2.40E + 02	4.14E-04
21	3.28E + 02	2.80E + 02	5.82E-04
22	3.28E + 02	3.20E + 02	7.87E-04
23	3.28E + 02	3.60E + 02	8.51E-04
24	3.28E + 02	4.00E + 02	1.03E-03
25	3.38E + 02	1.20E + 02	3.31E-05
26	3.38E + 02	1.60E + 02	9.09E-05
27	3.38E + 02	2.00E + 02	2.98E-04
28	3.38E + 02	2.40E + 02	4.81E-04
29	3.38E + 02	2.80E + 02	6.77E-04
30	3.38E + 02	3.20E + 02	8.89E-04
31	3.38E + 02	3.60E + 02	1.08E-03
32	3.38E + 02	4.00E + 02	1.24E-03

## Methodology

### GPR

Gaussian process regression is one of the base models used. GPR, unlike other regression models, does not necessitate the specification of an exact fitting function. A multidimensional Gaussian distribution sampled at random points can be compared to field data ^23,24^.

The target *y* is simulated as $f\left( {\varvec{x}} \right)$ for a collection of n-dimensional instances $D = \left\{ {\left( {{\varvec{x}}_{i} ,y_{i} } \right){|}i = 1, \ldots ,n} \right\}$, where ${\varvec{x}}_{i} \in R^{d}$ is input data point and $y_{i} \in R$ is the output vector.

1 $$y = f\left( {\varvec{x}} \right)$$

The GP is declared using *f(x)*, which is an implicit function illustrated as a collection of random variables:

2 $$f\left( {\varvec{x}} \right) \sim GP\left( {m\left( {\varvec{x}} \right),{\mathbf{K}}} \right)$$

In the above equation, *K* denotes any covariance defined by kernels and their corresponding input values and *m(x)* is the mean operator.

### Decision Tree

Trees are a fundamental data structure in a variety of AI contexts. An ML technique known as decision trees (DTs) is normally usage to measure the data. It is possible to utilize a decision tree to solve different estimation issues. To build a basic decision tree, you need internal nodes (which makes decision with query input features), edges (which return results and transmit them to children), and terminal or leaf nodes (which return results and send them to children) (that make decision on final output)^25,26^.

The root node is a special and unique node in the DT, which treats each dataset feature as a hub or node. To demonstrate how the tree model works, we start with a single node and work our way down the tree (output). Until a terminal node is found, this strategy will be tweaked and refined. The DT's forecast or outcome would be the terminal node^18,27,28^. The most useful algorithms for decision tree induction are CART^28^, CHAID^25^, C4.5, and C5.0^29^.

### ADABOOST

Multiple base predictors can be combined to create an ensemble learning-based model, which outperforms a single predictor. By altering the weight distribution of samples, Freund and Schapire^17^ proposed the AdaBoost algorithm for enhancing the accuracy of weak learners. Because of its advantages, this method has become increasingly popular^30,31^.

As the “AdaBoost” name implies, this technology adaptively enhances base predictors, enabling them to address complicated issues. One of the symptom for theamicability of basic models is that they have good generalization properties due to their simple structure. But despite the fact that they are easy to use in real-world situations, their architecture is severely biased, therefore they cannot handle complex jobs.

The Adaboost algorithm from Hastie et al.^32,33^ is mostly demonstrated in the following steps.

1. Set weights for data points:

3 $$\omega_{i} = \frac{1}{N} , i \in \left\{ {1, \ldots , N} \right\}$$

2. Set Number of base estimators as M.
3. For b from 1 to M:

(a) Develop a learner G_b_(x) using the weights $\omega_{i}$.

4 $$({\text{b}})\,\,\,err_{b} = \frac{{\mathop \sum \nolimits_{i = 1}^{N} \omega_{i} I\left( {y_{i} \ne G_{b} \left( {x_{i} } \right)} \right)}}{{\mathop \sum \nolimits_{i = 1}^{n} \omega_{i} }}$$

5 $$({\text{c}})\,\,\,\,\,\alpha_{b} = \log \left( {\frac{{1 - err_{b} }}{{err_{b} }}} \right)$$

6 $$({\text{d}})\,\,\,\,\,\,\omega_{i} \leftarrow \omega_{i} .\exp \left( {\alpha_{b} .I\left( {y_{i} \ne G_{b} \left( {x_{i} } \right)} \right)} \right), i = 1, \ldots ,N$$

4. Final Output:

7 $${\text{G}}\left( {\text{x}} \right) = sign \left( {\mathop \sum \limits_{b = 1}^{M} \alpha_{b} G_{b} \left( x \right)} \right)$$

In the previous procedure, the quantity of data vectors and the number of iterations are *N* and *M*, respectively. The estimator that passes *b* over the data is *G*_*b*_*(x)*. Building a prediction model (Base model) can be done in a variety of ways, but the most frequent is to employ stumps or very short trees. The operator I is set to 0 if the logical correlation is false and to 1 if the correlation is true, as shown by the indicator variable^34–36^.

## Results

Important hyper-parameters of selected models were first tuned applying the search grid method to assess the efficacy of the approaches described in this study. The resultant models were then examined using three distinct criteria, as specified below: MAE, MAPE, and R-score^37,38^:

8 $$\begin{array}{*{20}c} {{\text{MAE ERROR}} = \frac{1}{{\text{n}}} \times \mathop \sum \limits_{i = 1}^{{\text{n}}} \left| {{\hat{\text{y}}}_{{\text{i}}} - {\text{y}}_{{\text{i}}} } \right| } \\ \end{array}$$

9 $${\text{MAPE ERROR}} = \frac{1}{{\text{n}}} \times \mathop \sum \limits_{i = 1}^{{\text{n}}} \left| {\frac{{{\hat{\text{y}}}_{{\text{i}}} - {\text{y}}_{{\text{i}}} }}{{{\text{y}}_{{\text{i}}} }}} \right|$$

The third regression performance metric in our research is R^2^ score. The R^2^-Score is used on a regression line to determine how close the estimated amounts are to the true (expected) amounts.

10 $$\begin{array}{*{20}c} {{\text{R}}^{2} - score = 1 - \frac{{\mathop \sum \nolimits_{{\text{i = 1}}}^{{\text{n}}} \left( {{\text{y}}_{{\text{i}}} - {\hat{\text{y}}}_{{\text{i}}} } \right)^{2} }}{{\mathop \sum \nolimits_{{\text{i = 1}}}^{{\text{n}}} \left( {{\text{y}}_{{\text{i}}} - {\upmu }} \right)^{2} }}} \\ \end{array}$$

*μ* indicates the mean of the expected data^39^.

**Figure 1:**
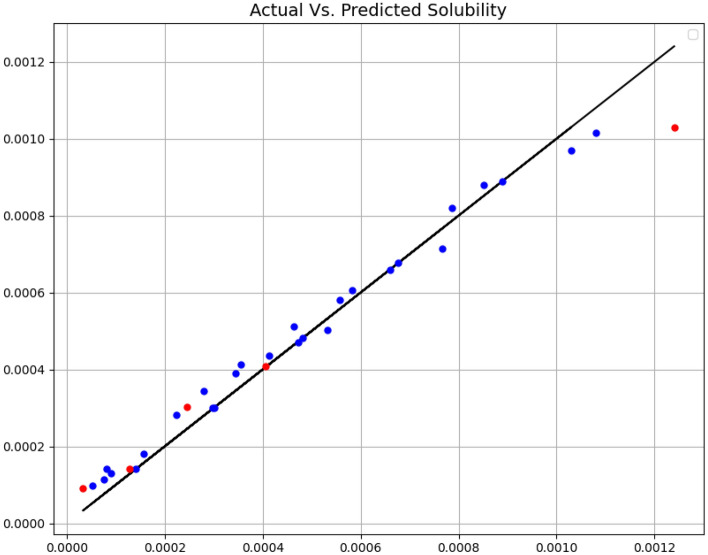
Expected and estimated values (ADA + DT).

**Figure 2:**
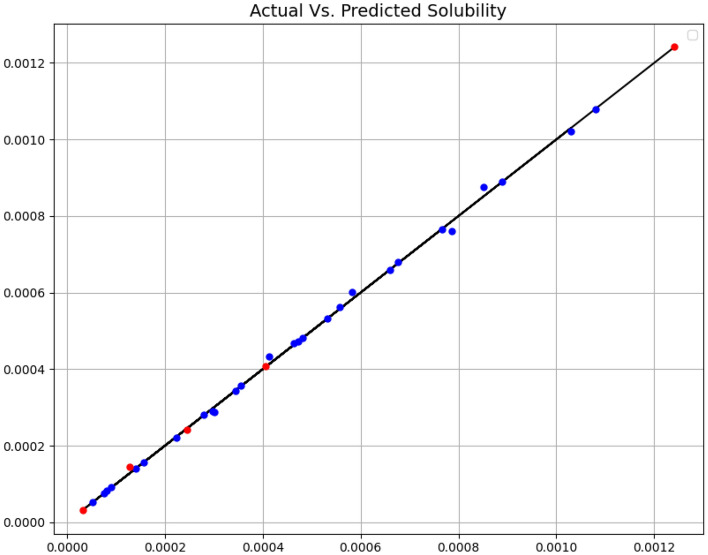
Expected and estimated values (ADA** + **GPR).

In Figs. 1 and 2, the ADA + DT and ADA + GPR models are analyzed in terms of expected values and estimated values, respectively. The blue dots are the estimated values with the training samples and the red dots with the test data. The distance from the expected data line is important to us. Also, the numerical results of the three criteria mentioned above are explained in Table 2. Based on results, the ADA + GPR model has passed almost all the points of the training data. But despite this fact, we can say that the obtained model has no overfitting problem because the red dots, which are test data and have not been included in the training phase, are also close to the expected values.

**Table 2:** Final Model Results.

Models	MAE	R^2^	MAPE
ADA + DT	6.806E-05	0.980	4.511E-01
ADA + GPR	5.024E-06	0.998	3.929E-02

**Figure 3:**
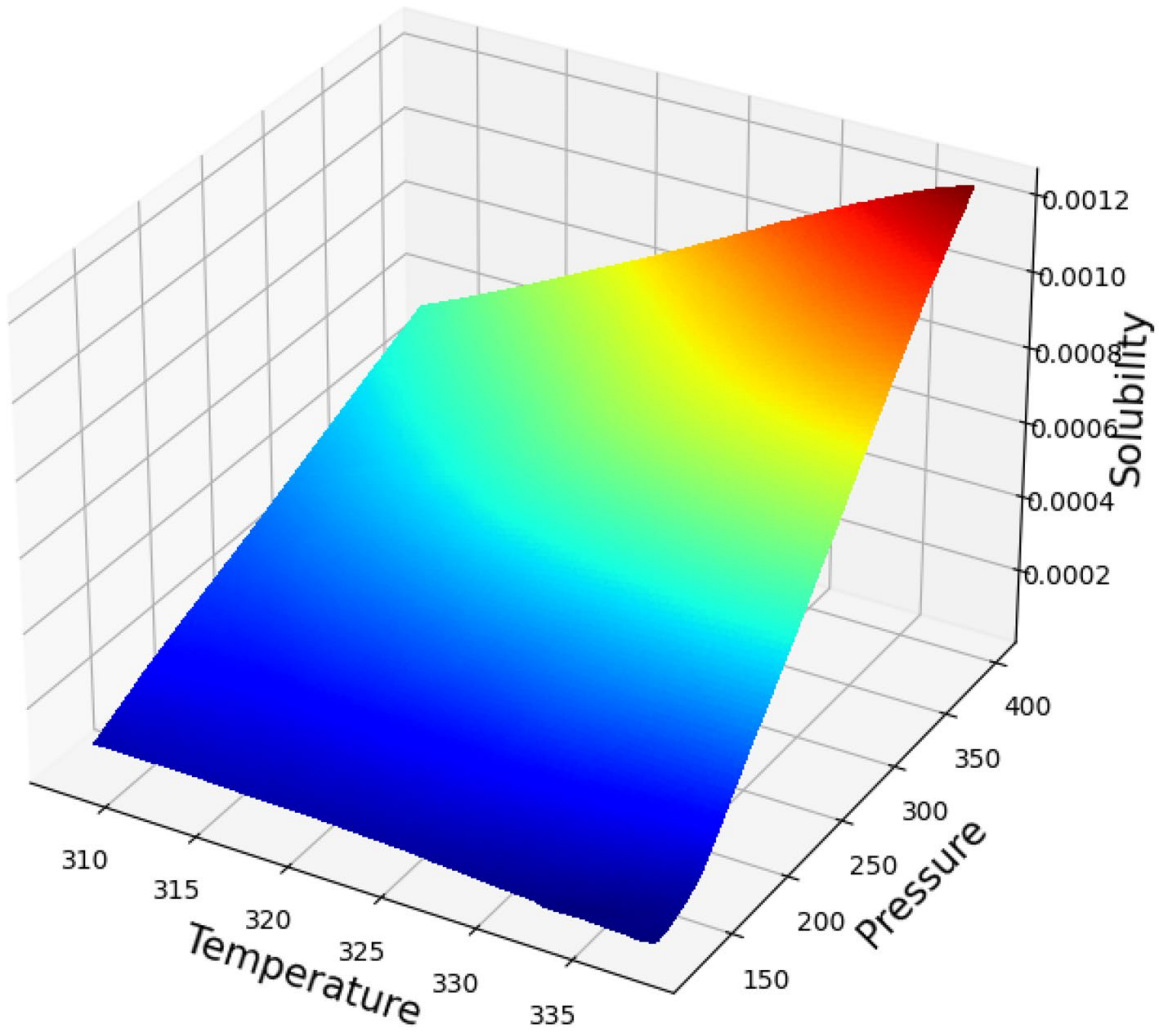
prediction surface in final ADA + GPR.

**Figure 4:**
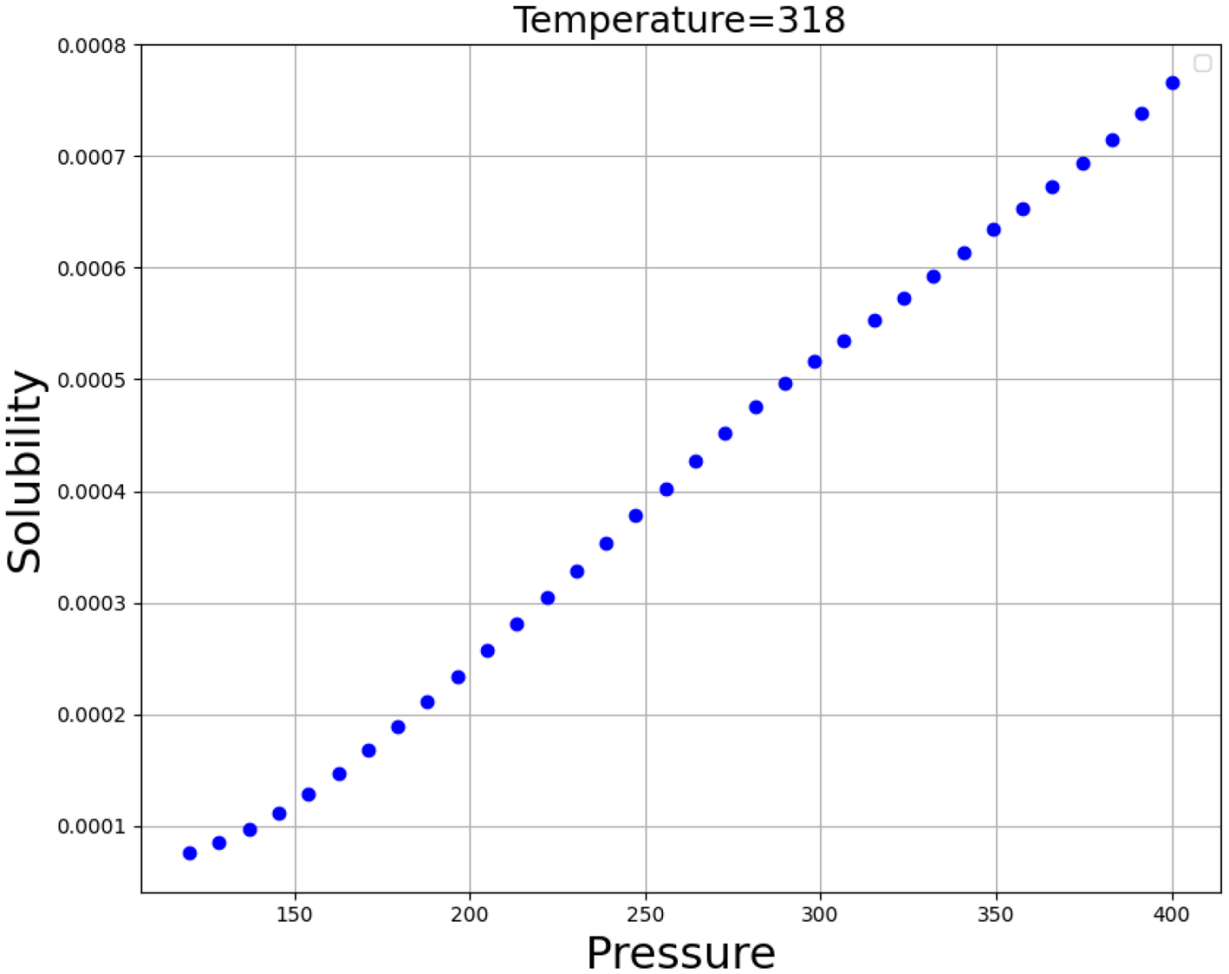
Trends for Pressure.

**Figure 5:**
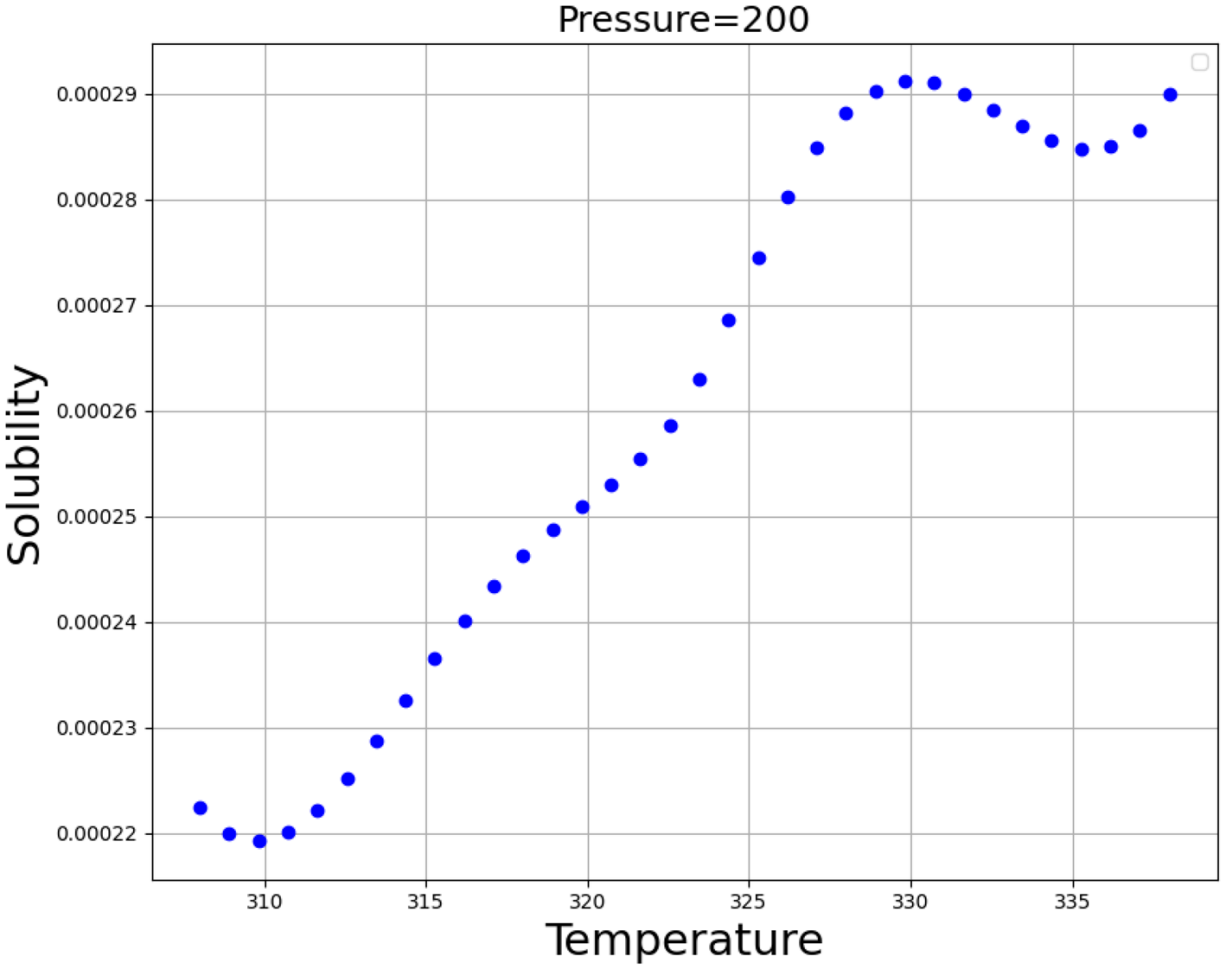
Trends for Temperature.

Figure 3 shows the simultaneous impact of the pressure and temperature as inputs the only output (Oxaprozin solubility). This diagram shows that increasing both inputs generally increase the output value. By keeping each of the two input parameters constant and changing the other parameter, we obtained two-dimensional Figs. 4 and 5, which confirms this fact. Figure 4 illustrates the influence of pressure and Fig. 5 demonstrates the impact of temperature on the solubility value of Oxaprozin. To analyze the diagrams, the effects of pressure and temperature on the solubility of drug must be considered. It is conspicuous from the graphs that whenever the temperature value improves, the molecular compaction in the SCCO_2_ system increases, which consequently eventuates in enhancing the solvating power of solvent and thus, increasing the solubility of Oxaprozin^40^. Figure 4 proves nearly 8 times enhancement in the solubility value of Oxaprozin by enhancing the pressure from 110 to 410 bar.

About temperature, the presence of opposite impacts on two competing parameters makes the analysis difficult. Increasing the temperature, the sublimation pressure of SCCO_2_ system increases that positively encourages the Oxaprozin solubility. On the other hand, increase in temperature deteriorates the density of solvent that results in reducing the solubility of drug. To evaluate simultaneous impact of these parameters, cross-over pressure (CP) must be considered. At pressure values lower than CP, density reduction possesses stronger effect than sublimation pressure increases and therefore, when the temperature increases the solubility of Oxaprozin in SCCO_2_ fluid reduces. At pressure values greater than CP, sublimation pressure increment has greater impact than density reduction and therefore, when the temperature increases the solubility of Oxaprozin in SCCO_2_ fluid considerably improves. This analysis agrees with similar papers^10^. The optimal values, which should therefore be approximately the upper limit of both inputs, are also shown in Table 3, which are the same as the maximum values.

**Table 3:** Optimal Values.

Temperature (K)	Pressure (bar)	Solubility
3.38E + 02	4. 0E + 02	0.001241

## Conclusion

In current years, increasing the solubility values of different commonly employed drugs using green solvents is an attractive field of study in pharmaceutics. SCCO_2_ has been recently introduced as a promising alternative for organic solvents because of having valuable features such as high efficacy, inflammability, and low toxicity. In this study, two base models (weak estimators) were used and boosted with Adaboost methods with the aim accurate prediction of Oxaprozin solubility in SCCO_2_ system. Decision tree (DT) and Gaussian process regression are two of these models (GPR). We optimized these models' hyperparameters and evaluated them using standard metrics. The MAE error rate, R^2^-score, and MAPE of boosted DT are 6.806E-05, 0.980, and 4.511E-01, respectively. Furthermore, boosted GPR has an R^2^-score of 0.998, MAPE error of 3.929E-02, and MAE error rate of 5.024E-06. As a result, ADA + GPR was chosen as the best model, with the following best values: (T = 3.38E + 02, P = 4.0E + 02, Solubility = 0.001241).

## Data Availability

All data are available within the published paper.
